# Decoding the brain-machine interaction for upper limb assistive technologies: advances and challenges

**DOI:** 10.3389/fnhum.2025.1532783

**Published:** 2025-02-06

**Authors:** Sutirtha Ghosh, Rohit Kumar Yadav, Sunaina Soni, Shivangi Giri, Suriya Prakash Muthukrishnan, Lalan Kumar, Shubhendu Bhasin, Sitikantha Roy

**Affiliations:** ^1^Department of Physiology, All India Institute of Medical Sciences, New Delhi, India; ^2^Department of Biomedical Engineering, National Institute of Technology, Raipur, India; ^3^Department of Applied Mechanics, Indian Institute of Technology Delhi, New Delhi, India; ^4^Department of Electrical Engineering, Bharti School of Telecommunication, New Delhi, India; ^5^Yardi School of Artificial Intelligence, Indian Institute of Technology Delhi, New Delhi, India; ^6^Department of Electrical Engineering, Indian Institute of Technology Delhi, New Delhi, India

**Keywords:** EEG, voluntary movement, movement related cortical potential, event-related desynchronization/synchronization, human-machine interaction

## Abstract

Understanding how the brain encodes upper limb movements is crucial for developing control mechanisms in assistive technologies. Advances in assistive technologies, particularly Brain-machine Interfaces (BMIs), highlight the importance of decoding motor intentions and kinematics for effective control. EEG-based BMI systems show promise due to their non-invasive nature and potential for inducing neural plasticity, enhancing motor rehabilitation outcomes. While EEG-based BMIs show potential for decoding motor intention and kinematics, studies indicate inconsistent correlations with actual or planned movements, posing challenges for achieving precise and reliable prosthesis control. Further, the variability in predictive EEG patterns across individuals necessitates personalized tuning to improve BMI efficiency. Integrating multiple physiological signals could enhance BMI precision and reliability, paving the way for more effective motor rehabilitation strategies. Studies have shown that brain activity adapts to gravitational and inertial constraints during movement, highlighting the critical role of neural adaptation to biomechanical changes in creating control systems for assistive devices. This review aims to provide a comprehensive overview of recent progress in deciphering neural activity patterns associated with both physiological and assisted upper limb movements, highlighting avenues for future exploration in neurorehabilitation and brain-machine interface development.

## 1 Introduction

### 1.1 Background

Human movements are intricately organized through synergies, influenced by affordances, and characterized by tension distributions within bio-tensegrity structures (Singh et al., 2018; Franchak et al., 2010). Recent advancements in Brain-Machine Interfaces (BMIs) underscore their critical role in enhancing motor rehabilitation by precisely decoding motor intentions for voluntary movements. BMIs are primarily designed to restore or augment goal-directed and voluntary movements. Voluntary motor actions involve a top-down intention to initiate movement, but effective voluntary control also relies on selectively utilizing bottom-up sensory feedback to inform and guide these actions (Scott, 2016). It is crucial to develop assistive BMIs that integrate both top-down intention and bottom-up sensory feedback. Recent literature has advanced the current understanding of decision-making processes involved in goal-directed movements. Mirabella (2014) proposed that actions and their inhibition are tightly coupled within overlapping neural circuits, challenging the classical view of distinct regions for movement and stopping actions. These insights highlight the complex decision-making networks underlying voluntary motor control, which are essential for refining assistive technology systems designed to interface with human intentions.

The human brain executes motor functions through complex interactions among various brain regions, including the cerebellum, basal ganglia, and motor-related cortical areas like the primary motor cortex (PMC) (Caligiore et al., 2017). Basal ganglia and cerebellum are essential for controlling the motor system, modulating the activity of premotor cortex (PrMC), supplementary motor area (SMA), and PMC (Rocha et al., 2023). Cerebellum compares planned actions to executed ones, providing feedback to refine motor movements and correct errors (Antonioni et al., 2024). Basal ganglia are involved both in coordinating the motor plan and inhibiting goal-directed movements. This is achieved by integrating cortical inputs and fine-tuning motor plans via direct and indirect pathways, by modulating the dopaminergic neurons (Lanciego et al., 2012; Parent, 2012). Characteristically, voluntary movements commence with neural signals in the motor cortex, transmitting from the central nervous system (CNS) to the peripheral nervous system (PNS), ultimately triggering muscle contractions (Ullsperger et al., 2014). Specific and coordinated muscle activation patterns are necessary for precise action execution (Zhao et al., 2023).

Human movements have often been characterized by kinematic and kinetic attributes (Jones and Lederman, 2006). Kinematic parameters pertain to the motion and spatial characteristics of movement and are also referred to as “high-level control.” The essential kinematic parameters of movement are the location, direction, velocity, and acceleration, which result in the desired trajectory. In contrast, kinetic parameters, often known as “low-level control,” are related to the control of individual muscles and forces. Furthermore, an individual can employ numerous trajectories to execute a goal-oriented movement. Previous studies have explored the representation of independent and/or integrated kinematic and kinetic features of upper limb movements in the sensorimotor cortex (Branco et al., 2019; Zhou et al., 2022; Teka et al., 2017; Dokkum et al., 2017). However, understanding how an individual’s brain finds the optimal solution to carry out a voluntary task remains one of the biggest scientific challenges.

Understanding the transformation of neural information into voluntary movement is pivotal to restore motor functions in neurological diseases such as cerebral stroke, spinal cord injury and so on. Recent developments of assistive technologies coupled with BMI underscore the significance of decoding user’s motor intentions for effective control (Sambhav et al., 2022; Wang et al., 2023). In the context of wearable robotics, where the human and the device are physically connected, synchronous movement is crucial. This synchronization could be reflected in the neural correlates, which might be utilized to optimize assisted motor movements (Varghese et al., 2018).

Even though invasive BMIs have a higher signal-to-noise ratio, they pose adverse effects due to surgery and could become incompatible in the long run because of tissue reactivity (Hochberg et al., 2006). On the other hand, electroencephalography (EEG) has proven to be an excellent non-invasive technique to explore the neurophysiological metrics like motor-related cortical potentials (MRCP) (Pfurtscheller and Aranibar, 1979; Kornhuber and Deecke, 2016) and event-related desynchronization/synchronization (ERD/S) to decode upper limb movements (Jochumsen et al., 2017; Tang et al., 2016). Error-related potentials (ErrPs), another important parameter, are a subset of event-related potentials (ERPs) and serve as indicators of instances where a wearable robotic device deviates from human expectations (Chavarriaga et al., 2014; Spuler and Niethammer, 2015; Lopes-Dias et al., 2019). ErrPs enable adjustments in the machine’s responses, aligning them with human preferences and enhancing the overall interactive experience (Ullsperger et al., 2014; Omedes et al., 2018).

Neural plasticity refers to the brain’s ability to adapt and reorganize itself in response to experience (Xu et al., 2014; Rossini et al., 2012). Long-term application of EEG-based assistive devices has been documented to induce neural plasticity. The concept of neural plasticity opens avenues for the development of more effective and personalized assistive technologies. In the context of motor rehabilitation for stroke patients, neurofeedback therapy (NFT) based on assistive technology has been proven to be beneficial when integrated with traditional therapies (Rayegani et al., 2014).

For self-paced movements, significant ERD of alpha and beta frequency has been documented in the contralateral motor cortex, particularly preceding the movements (−1,000 ms) (Deiber et al., 2012). Conversely, in the context of cue-based actions, bilateral alpha ERD reaches its maximum over the parieto-occipital areas during the planning phase prior to the movement (−1800 ms to −2000 ms) (López-Larraz et al., 2014). Studies on MRCP patterns have shown the involvement of motor areas and posterior parietal lobule in specific goal-oriented tasks (Pereira et al., 2017; Sburlea et al., 2021). This spatio-temporal specificity highlights the intricate orchestration of neural dynamics during different types of motor tasks. Nevertheless, there is a lack of consensus on the interpretation of these findings, thus indicating a need for further investigation.

Although extensive research has been done on the neural correlates of upper limb movements and kinematics, less is known about how these correlates are modulated during externally assisted movements. Furthermore, little is known about how biomechanical changes alter brain activity. Hence, a large-scale review of the published literature is required to answer the following questions and reach coherent conclusions.

(i) Which are the neural correlates predictive of upper limb motor intention and kinematics?
(ii) What are the effects of external assistance on the neural correlates of upper limb movement?
(iii) What are the effects of changes in biomechanical characteristics on brain activity?

### 1.2 Objectives

The primary objective of this study is to conduct a comprehensive review of the existing literature to evaluate the neural correlates predictive of upper limb motor intention and kinematics. This study also investigates the impact of external assistance on these correlates during upper limb movement. The secondary objective of this study is to investigate the effects of changes in biomechanical parameters on brain activity during upper limb movement. This work attempts to provide a thorough understanding of the interactions between neural correlates, external assistance, and biomechanical factors by methodically integrating available findings from relevant research.

## 2 Methodology

### 2.1 Transparency and openness

This review was conducted following the Preferred Reporting Items for Systematic Reviews and Meta-Analyses (PRISMA) guidelines (Page et al., 2021). However, the review was not pre-registered, and no review protocol was developed before initiating the review process.

### 2.2 Review planning and search strategy

**Figure 1:**
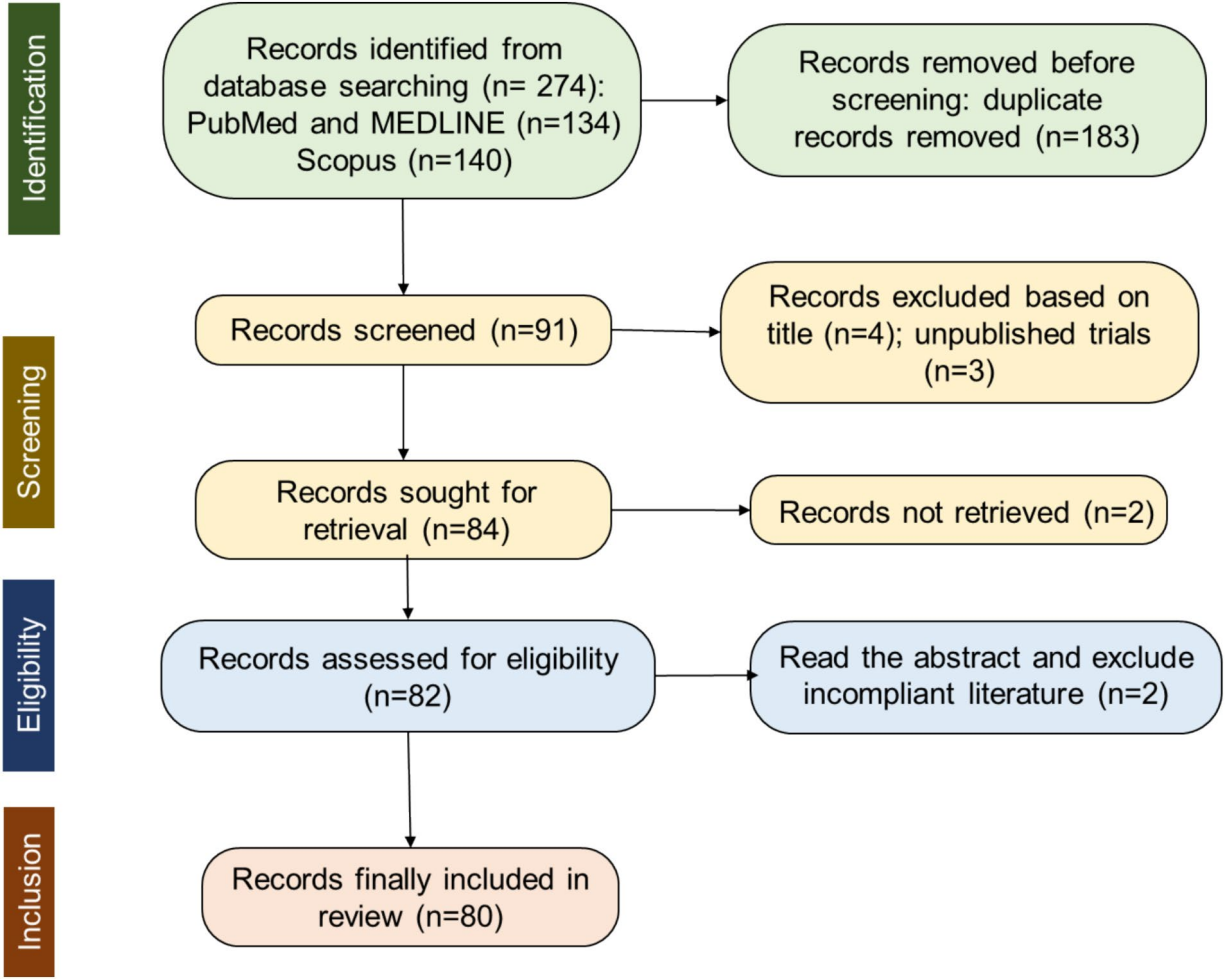
Flowchart of PRISMA record selection process.

The database search was conducted in October 2024 using PUBMED, MEDLINE, and Scopus by two independent reviewers. Articles were screened based on titles, abstracts, or keywords that included “Upper limb movement” AND “EEG” AND (“Assistance OR Kinematics”). The selection process is illustrated in Figure 1, which shows the PRISMA flowchart.

An initial limited search of PUBMED, MEDLINE, and Scopus were undertaken to identify articles on the topic in October 2024. The words contained in the titles and abstracts of significant articles, and the index terms used to describe them were used to formulate a full search strategy (Figure 1). This consisted of specific keywords joined together by Boolean operators “AND” and “OR.” The first search criteria were restricted studies on “Upper limb movement” AND “EEG” and the second focused on “Upper limb movement” AND (“Assistance” OR “Kinematics”) (using all the related terms found in the initial search and/or known to be relevant). Thus, the strategy considered synonyms and related terms, and used Boolean operators. The search strategy, including all identified keywords and index terms, were adapted for each included database and/or information source. The reference list of all included sources of evidence were screened for additional studies.

### 2.3 Eligibility criteria

The search was restricted to studies involving human participants. Studies that had full-text articles and published exclusively in English were included. Articles published in peer-reviewed journals from 1 January 2004 onwards were considered eligible. Eligible studies had to manipulate at least one of the following: upper limb movements or upper limb assistance, and they needed to investigate neural correlates and kinematic changes.

Studies were excluded if they were not published in English or were published before the year 2004. Additionally, articles not having full texts available or lacking a DOI were excluded. We also did not include studies that did not focus on the adult population or were only available as preprints. Our focus was on research exploring the neural correlates of external assistance for upper limb movements or exoskeleton use. Studies involving lower-limb with/without assistance were excluded.

### 2.4 Data collection and analysis

#### 2.4.1 Selection of studies

Two reviewers (SG, RKY) independently screened the search results. This dual-reviewer approach was maintained throughout all the review stages. Two other reviewers (SS, SG) evaluated the studies which were screened by the independent reviewers and suggested changes in the screening patterns and techniques to narrow down the search strategy.

Studies that (1) examined neural correlates of upper limb motor movements, (2) had upper limb movements with and without external assistance and (3) used brain activity measurement methods to evaluate neural correlates during motor tasks, (4) studied changes in biomechanical parameters during upper limb motor movements, were considered eligible. The reviewers and the corresponding author (SPM) then independently read the titles and the abstracts of screened studies and eliminated the irrelevant studies. Full text for the remaining studies was obtained and, according to the previously mentioned inclusion and exclusion criteria, they were independently ranked as relevant, potentially relevant, and irrelevant. Since the authors are from different backgrounds, their insight about specific domains has been beneficial in framing the structure of the methodology. The manuscript is written by the first four authors which was reviewed and proofread by all the other authors.

In cases where there was a disagreement between reviewers regarding the relevance, quality, or interpretation of an article, the following steps were taken:

- The authors discussed the article in question collectively in regular meetings to clarify differing perspectives.
- If the disagreement persisted, the corresponding author (SPM) was consulted to provide an independent opinion.
- Decisions were made by consensus wherever possible. In rare cases where consensus could not be reached, the majority opinion was followed.

#### 2.4.2 Assessment of risk of Bias in included clinical studies

Since systematic biases can lead to either an underestimation or an overestimation of the true intervention effect (Higgins et al., 2008), it is necessary to evaluate the risk of bias in clinical trials in order to reduce the likelihood of making incorrect decisions regarding treatment effects (Gluud, 2006). According to Sterne et al. (2016), this study made use of the ROBINS-I tool (Risk Of Bias In Non-randomized Studies–of Interventions) to evaluate twenty clinical studies included in this review. The core idea behind ROBINS-I is to compare the bias risks of the current study under evaluation with those of a target RCT that is hypothetically carried out on the same participant group, even if this RCT may not be practical or morally acceptable (Schünemann et al., 2018).

The ROBINS-I tool includes the evaluation of seven domains through which bias might be introduced into a non-randomized study: (1) bias due to confounding; (2) bias in classification of interventions: (3) bias in selection of participants into the study; (4) bias due to deviations from intended interventions; (5) bias due to missing data; (6) bias in measurement of outcomes (or detection bias); (7) bias in selections of the reported results. To make risk assessments easier, the ROBINS-I tool has a set of signalling questions in each domain. Low, moderate, serious, and critical risk are the categories for risk of bias judgements. First, the risk of bias is evaluated for each domain, and then the study as a whole. Three authors independently assessed risk of bias of the included studies (SG, RKY, SPM), with disagreements between reviewers resolved through discussion between all authors.

## 3 Results

### 3.1 Retrieved papers

For more details on the search and screening process, refer to the PRISMA flow diagram in Figure 1. The initial search yielded a total of 274 citations: 134 from PubMed and MEDLINE, and 140 from Scopus. Before the screening procedure, a total of 183 duplicates were removed, and 91 articles remained. Based on the title and the abstract of the articles, 7 were excluded, with 84 full-text articles to be retrieved and assessed for eligibility. 2 articles out of these could not be retrieved. Thus, 82 full-text articles were assessed for eligibility. The content of these studies was inspected to further determine their eligibility according to the predefined criteria. Two of them were excluded for not being peer-reviewed yet, resulting in a final selection of 80 studies that met all eligibility criteria.

This review mainly considers peer-reviewed experimental studies within the fields of human neuroscience, BMI, robotic devices, and neuro-rehabilitation in healthy and neurological patients. As the primary aim of this review is to shed light on the predictive neural correlates of upper limb motor intention and kinematics, it mainly focuses on studies based on neurophysiological techniques like EEG, electromyography (EMG), and kinematics. Additionally, systematic reviews/meta-analyses which met the inclusion criteria were also scrutinized for useful evidence, depending on their research questions.

### 3.2 Assessment of risk of bias in included clinical studies

**Figure 2:**
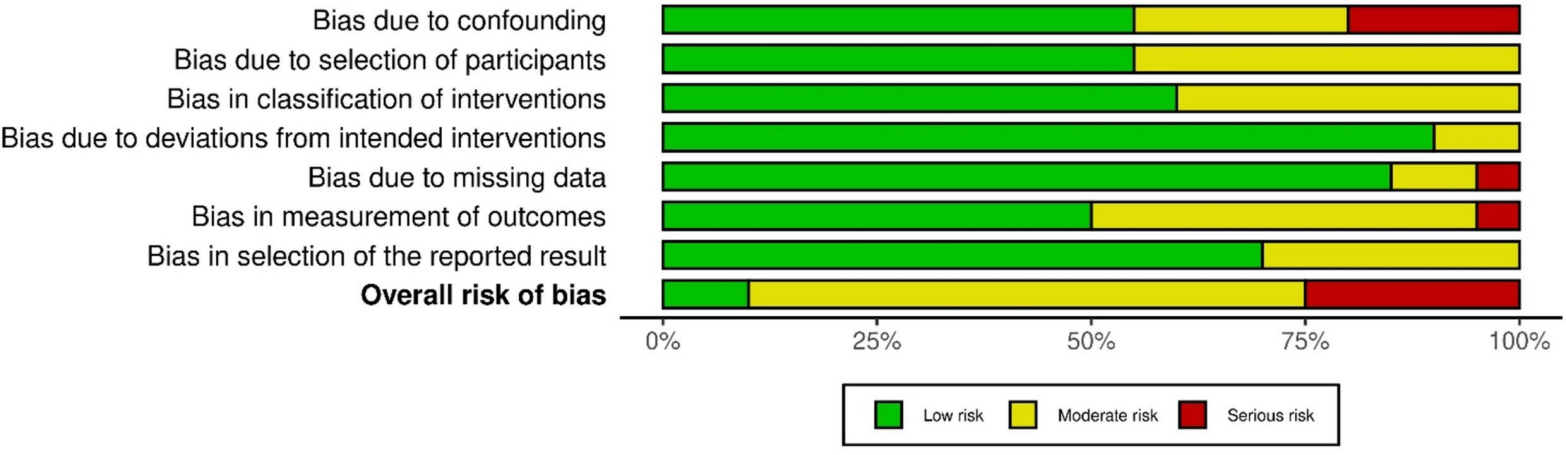
Risk of bias presented as percentages across all included clinical studies.

Risks of bias represented as percentage across all included clinical studies are shown in Figure 2 and Appendix 1. Following the ROBINS-I tool, the risks of bias have been classified as follows:

*Confounding bias:* Most studies exhibited a low or moderate risk of confounding bias. However, exceptions included Bhagat et al. (2016, 2020), Gu (2009), and Ottenhoff et al. (2023). These studies either did not control for confounders or did not conduct a pre- vs. post-intervention data analysis.

*Selection Bias:* The risk of bias in participant selection was low in eleven studies (Bhagat et al., 2016; Wodlinger et al., 2015; Wilkins et al., 2017; Tang et al., 2023; Cantillo-Negrete et al., 2021; Rayegani et al., 2014; Bhagat et al., 2020; Gandolfi et al., 2018; Gu, 2009; Hochberg et al., 2006; Hortal et al., 2015; López-Larraz et al., 2014; Mondini et al., 2024; Ofner et al., 2019; Ottenhoff et al., 2023; Pichiorri et al., 2023; Pulferer et al., 2022; Rohm et al., 2013; Serino et al., 2022; Várkuti et al., 2013), as these studies followed specific inclusion criteria and enrolled all eligible participants. However, nine studies had a moderate risk of selection bias due to the absence of follow-up data.

*Intervention Classification Bias:* Twelve studies had a low risk of bias, as they collected data during the intervention phase, ensuring all participants followed the same protocol, with outcome measures recorded immediately. In contrast, eight studies did not explicitly describe the intervention, leaving uncertainty about the data collection before the intervention.

*Deviation from Intended Intervention Bias:* Two studies (Gu, 2009; López-Larraz et al., 2014) showed deviations from the intended intervention. In Gu (2009), one participant performed the motor task with both wrists, potentially introducing bias. In López-Larraz et al. (2014), although two groups were mentioned, no comparative analysis between them was conducted.

*Missing Data Bias:* Only one study (Rayegani et al., 2014) exhibited a high risk of bias due to participant dropout post-intervention. In two studies (Hortal et al., 2015; Várkuti et al., 2013), the risk was moderate, as some participants’ data were excluded from the final analysis. All other studies reported complete datasets.

*Outcome Measurement Bias:* López-Larraz et al. (2014) was classified as having a serious risk of bias, as the absence of inter-group comparisons could have affected the outcome measurements. Nine studies had a moderate risk, as they did not specify whether assessors were blinded to the intervention during outcome assessment. The remaining studies had minimal measurement bias.

## 4 Discussion

### 4.1 Neural correlates predictive of upper limb motor intention and kinematics

**Figure 3:**
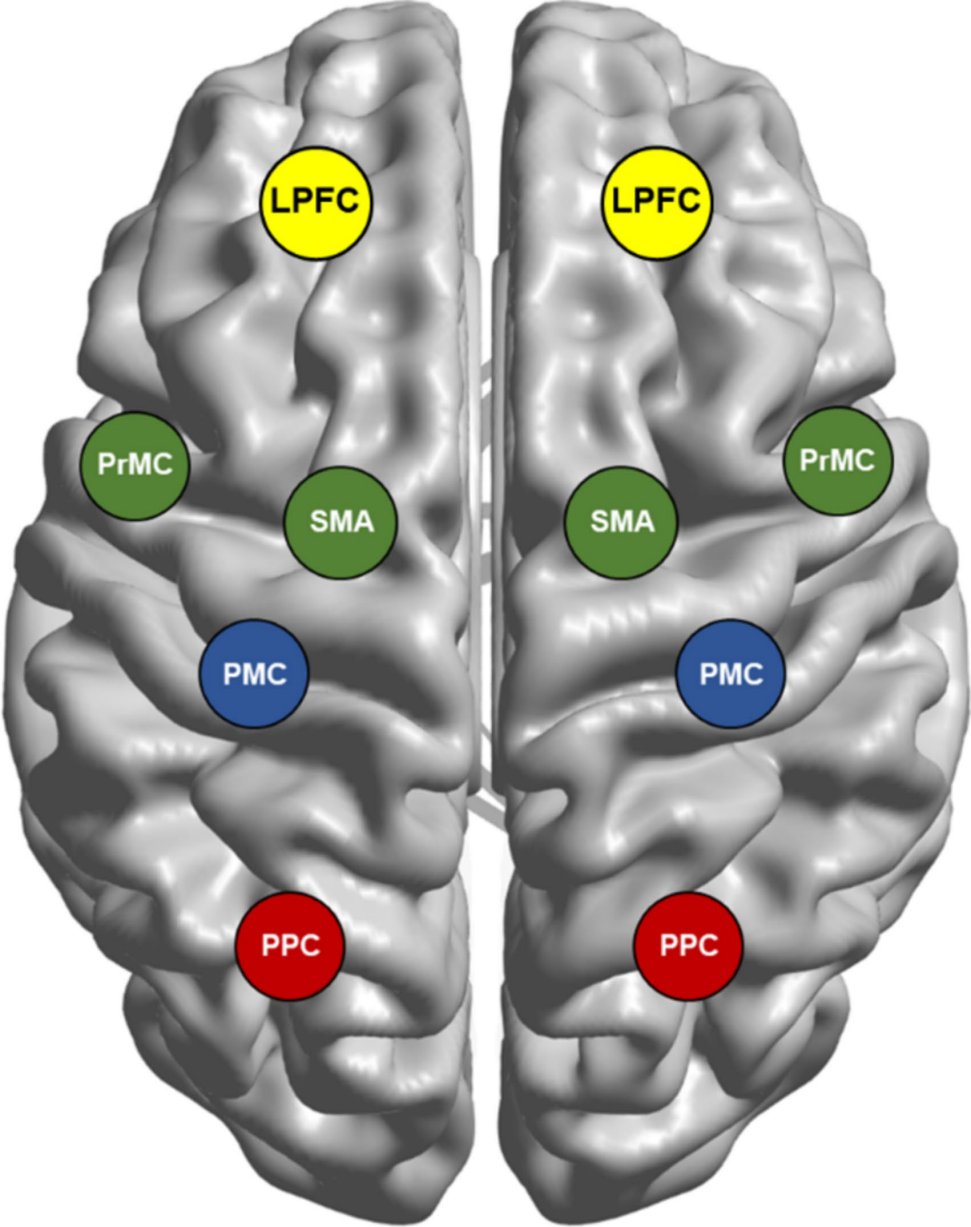
Brain network involved in movement planning and execution. The motor system has a hierarchical organization, with each level subserving distinct processes for a movement. First, the posterior parietal cortex (PPC) and premotor cortex (PrMC) form a sensory representation of the peripersonal space, while supplementary motor area (SMA)/pre-SMA assesses the goal-worthiness of movements. Second, the lateral prefrontal cortex (LPFC) receives the diverse information of both external cues and internal states to select appropriate goals. Subsequently, parietal reach region (PRR), PrMC, and primary motor cortex (PMC) choose the most suitable action, also forming a motor plan and guiding the movement. The final action evaluation involves inferior frontal gyrus (IFG), LPFC, pre-SMA, PrMC, and PMC. Further, the PMC controls action execution by managing muscle force and direction, ensuring precise movement. These cortical motor areas are interconnected through complex patterns of reciprocal, convergent, and divergent projections, rather than simple serial pathways. While serial processing in the motor network provides insight, evidence indicates that many of these processes can also occur in parallel.

Discovering how the brain encodes motions is essential for designing efficient control mechanisms for robotic arms and motor neuroprostheses. The motor system is organized in a hierarchical architecture based on the specific functions executed by the associated brain regions (Figure 3).

First, the formation of a sensory map of peripersonal space is subserved by the posterior parietal cortex (PPC) (Lindner et al., 2010) and PrMC (Holmes and Spence, 2004). SMA/pre-SMA is involved in the evaluation of worthiness of a goal-directed actions (Guida et al., 2023).

Second, lateral prefrontal cortex (LPFC) receives diverse information from environmental resources (e.g., numerosity, duration, distance) from PPC (Genovesio et al., 2014) and several other regions to synthesize information to select the more appropriate goal relevant to the given context.

Third, left dorsal premotor cortex (PrMd) (Klaes et al., 2011), PMC (Caligiore et al., 2017) and parietal reach region (PRR), a subregion of the PPC (Cui and Andersen, 2007), chooses the more appropriate action to achieve the goal selected by LPFC. For the selected action, the same substrates are responsible for the formulation of motor plan and guidance of movement execution.

Fourth, the final evaluation of selected motor action is processed in a large network of brain regions involving inferior frontal gyrus (IFG) and dorsolateral prefrontal cortex (DLPFC) (subregions of LPFC), pre-SMA, PrMd and PMC (Mirabella, 2014).

Finally, if the action selected passes the final evaluation process, PMC executes movement by controlling muscle force and direction (Makino et al., 2016).

In addition to the brain regions of the motor system vide supra, cerebellum and basal ganglia contribute significantly to modulate and precisely fine tune voluntary movements. The immense density of neuronal circuits known as motion adjustment loops in cerebellum is responsible for maintaining balance, posture, fine tuning of movements and motor learning (Rocha et al., 2023). The basal ganglia facilitate movement through the direct pathway, exciting the motor cortex via the thalamus to initiate planned actions (Parent, 2012). The indirect pathway inhibits competing motor actions by suppressing the thalamus, preventing unwanted movements (Lanciego et al., 2012).

Despite the existence of this hierarchical model of the cortical motor network involved in movement planning and execution (Figure 3), the neural mechanisms responsible for generating motor commands from the PMC remain unclear (Makino et al., 2016; Diedrichsen and Kornysheva, 2015). This understanding can be refined by considering more specific decision-making processes related to goal-directed actions. For instance, Mirabella (2014) outlined a framework suggesting that decision-making, such as determining whether to act or inhibit movement, occurs throughout the genesis, planning, and execution phases of goal-directed behavior (Mirabella, 2014). Furthermore, Cisek and Kalaska (2010) proposed a competitive process in the dorsal premotor cortex where multiple action plans are represented in parallel, and one is selected based on internal and external cues (Cisek and Kalaska, 2010). While substantial primate and human research has shed light on these processes, the identification of movement features for BMI control, namely kinematics (spatial and motion aspects) and kinetics (muscles and forces) in the sensorimotor cortex (SMC) and their role in producing precise movements are not completely understood (Branco et al., 2019).

Functional electrical stimulation (FES)-based prostheses have been used to restore movement in spinal cord injury (SCI) patients, but advancements in BMI technology offer a more natural and comfortable alternative (Rupp and Gerner, 2004; Rupp et al., 2015). BMI allows the decoding of movement intentions into control signals for robotic arms or neuroprosthetics to provide more accurate and effective control (Sambhav et al., 2022). To regulate neuroprostheses, the control systems rely on preparatory brain signals such as readiness potential (RP) and sensorimotor rhythms (SMR) linked to motor imagery (MI) movements (Libet et al., 1983: Kraeutner et al., 2016). While identifying preparatory brain signals is central to BMI control systems, the brain can still cancel a movement after these signals are initiated (Mirabella, 2021). Evidence indicates that movement cancellation is possible if the brain issues stop signals at least 200 ms before the movement begins, defining a “point of no return” (Schultze-Kraft et al., 2016). SMR-based BMIs have been shown to restore limb functions in tetraplegic participants suggesting that they have the potential to be used in rehabilitation (Wu et al., 2013). SMR-based BMIs have several drawbacks which include the need for repeated MI and less effectiveness to identify spatially distinct EEG patterns. Continuous decoding of movement trajectories from EEG data could be a potential solution to these challenges (Yong and Menon, 2015; Edelman et al., 2019).

MI refers to the mental representation or intention of executing a movement (Kraeutner et al., 2016). Cerebellum, PMC, and SMA are among the brain areas that are activated both during MI and motor execution (ME) (Meister et al., 2005; Lu et al., 2012). However, there are notable distinctions between MI and actual execution (Mizuguchi et al., 2016), such as reduced activation of the PMC during MI (Mizuguchi et al., 2017; Hétu et al., 2013). Both ME and MI can cause differences in neural activity in sensorimotor areas (Tang et al., 2016; Yang et al., 2022) (Table 1). Similarly, studies on motor attempt has also reported activation in central motor areas when SCI patients attempted various arm and hand movements (Ofner et al., 2019; Pulferer et al., 2022; Muller-Putz et al., 2022). Regional beta synchronization in the left hemisphere could distinguish between movements of the left and right hands in both MI and ME (Demuru et al., 2013). This pattern implies that activity in motor-related brain areas during both MI and ME increases with task difficulty.

**Table 1:** Predictive neural correlates of upper limb motor intention and kinematics.

References	Sample size, tools used	Movement studied	Observations
Tang et al. (2016)	4 healthy subjects, Mean age: 28 ± 2.6. EEG	Motor execution (ME) and motor imagery (MI) of left/right wrist extension	Alpha and beta ERD were observed over motor regions contralateral to the hand movements around 1–4 s after cue. Classification accuracy of decoder with the participants wearing the exoskeleton: ■ ME: 87.37% ± 3.06% ■ MI: 84.29% ± 2.11%
Yang et al. (2022)	12 healthy subjects, Mean age: 24.8 ± 2.0. EEG, fMRI	MI: Grasp., reach, handling, rotation	Supplementary motor area (SMA) and pre-cingulate gyrus (preCG) showed higher activation in all the MI movements. Highest activation in these areas were observed 1.5 s after voice cue during MI.
Bhagat et al. (2016)	10 stroke survivors, Mean age: 55 ± 9.2. EEG, EMG, Motion capture	Elbow flexion-extension	From MRCP amplitude modulations in primary motor cortex and post-central gyrus, the motor intent was predicted before movement onset: ■ Online detection (−66 ± 86 ms) ■ Offline detection (−723 ± 740 ms)
Ofner and Muller-Putz (2015)	9 healthy subjects, Mean age: 26.1 ± 4.3 years EEG	MI: Right arm extended movement (center—left—right—center), (center—top—bottom—center)	Decoding movements from EEG during MI, showed significant correlation in the premotor cortex (PrMC), primary motor cortex, somatosensory cortex and posterior parietal cortex. Absence of uniform weight pattern across subjects, suggesting that EEG decoding models require individual tuning.
Lu et al. (2012)	10 healthy subjects, Mean age: 27.9 ± 6.9. EEG, rTMS	Visually cued self-initiated finger movement	Application of 1 Hz rTMS at left dorsal premotor cortex (PrMd) augmented late contingent negative variation (CNV) amplitude over left central region 1.5 to 0.5 s before movement. Bereitschaftspotential remained unaffected by PrMd rTMS. This temporal delineation of cortical potentials, provides evidence of the role of the PrMd being critically involved in externally guided motor tasks.
Wodlinger et al. (2015)	1 tetraplegic patient, Age: 52 years. Intracortical microelectrode recording, BMI	Pinch, scoop, finger abduction, thumb opposition	Broad distribution of neural unit preferred directions across the 10D space highlights motor cortex underscoring the complex multidimensional encoding in BMI applications. Significantly, the majority of neural units exhibited tuning preferences across all ten dimensions of movement.
Fifer et al. (2014)	Subject 1: 55-year-old, Subject 2: 30-year-old. Modular prosthetic limb using intracranial EEG	Reach and grasp	The intracranial EEG (iEEG) site responsible for controlling reaching was found dorsally to the site responsible for controlling grasping. This spatial organization suggests a functional segregation within the motor cortex for different aspects of movement control. High gamma power amplification showed significant spatial selectivity relative to task-related cortical activity suggesting the effectiveness of iEEG as a source of BMI signal.
Roosink and Zijdewind (2010)	20 healthy subjects, Mean age: 25 ± 7. EMG, TMS	Simple and complex finger tapping sequences	Increased corticospinal excitability during active observation was reported compared to passive observation, visual, or kinesthetic imagery. First burst of TMS activity was seen in the FDI (First Dorsal Interosseous) during action execution varied from 47 to 689 ms after the start signal.
Zhou et al. (2022)	21 healthy subjects, Mean age: 24.62 ± 1.5. fNIRS, 3D motion capture	Single and bilateral finger movement	Contralateral activation in prefrontal cortex and motor cortex (t = 0.05 s), emphasized the lateralized control of unilateral movements Hand dominance and the complexity of motor tasks (in-phase vs. antiphase movements) influenced distinct brain activation.
Sosnik and Zur (2020)	9 healthy subjects, Mean age: 26.5 ± 3.5. EEG, 3D motion capture	Actual and imagined pointing movement to targets.	Brain regions for actual hand trajectories: Contralateral precentral gyrus (PMC and PrMC) ○ Postcentral gyrus (primary sensory cortex) ○ Contralateral posterior medial frontal cortex (SMA) Brain regions for actual trajectories of the shoulder: Parietal lobule.

EEG, Electroencephalography; PMC, Primary motor cortex; PrMC, Pre-motor cortex; SMA, Supplementary motor area; fNIRS, Functional Near Infrared Spectroscopy; rTMS, Repetitive transcranial magnetic stimulation; BMI, Brain machine interface; fMRI, Functional magnetic resonance imaging; EMG, Electromyography; ERD, Event related desynchronization; MRCP, Movement related cortical potential.

According to Hortal et al. (2015), MI based on scalp-recorded EEG makes it easier for users to operate assistive technologies. MI-BMI training has demonstrated promise in stroke therapy and neuronal recovery (Catrambone et al., 2018; Gandolfi et al., 2018). It is possible to optimize closed-loop EEG-based BMIs for stroke patients for long-term usage without having to recalibrate them (Bhagat et al., 2016) (Table 1). BMI systems controlled by hand MI could induce neural plasticity in stroke patients, which is correlated with an improvement in upper limb functions (Bhagat et al., 2020). While the EEG showed promise in predicting upper limb movements during MI, its effectiveness varied across subjects due to the absence of a consistent activation pattern, necessitating individualized tuning (Ofner and Muller-Putz, 2015) (Table 1).

BMIs utilize a structured approach for decoding brain signals, involving key stages such as signal processing, feature extraction, classification, and feedback loops. Initially, bio-signals such as EEG are processed to enhance signal to noise ratio, followed by the extraction of relevant features that represent brain activity patterns. These patterns are then classified into commands by decoding algorithms to operate external devices, with the cycle completed by providing user feedback. Extraction and classification of relevant features into commands are done using decoders such as linear discriminant analysis (LDA), naive Bayes, and support vector machines (SVM) for discrete robotic movements. Other methods include convolutional neural networks (CNNs), recurrent neural networks (RNNs) and its variant long short-term memory (LSTM), and deep neural networks (DNNs) (Ottenhoff et al., 2023). Then, there are autoencoders, and deep belief networks (DBN) which enhance end-to-end learning from raw signals, thus bypassing manual feature extraction (Yang et al., 2022). To decode continuous movement, deep learning methods are combined with the Kalman filter to enhance and optimize the abundant motor-related information found in intracortical signals. These decoders are extensively applied across offline analyses, real-time applications, and clinical trials (Dong et al., 2023).

The application of 1 Hz repetitive transcranial magnetic stimulation (rTMS) to the PrMd has been reported to augment the late contingent negative variation (CNV) amplitude over the left central region while the Bereitschaftspotential remained unaffected suggesting a crucial role of PrMd in cue-guided movements (Lu et al., 2012) (Table 1). Through invasive methods, reports have shown progressive neuronal recruitment in SMA over 1,500 ms before self-paced movements (Kornhuber and Deecke, 2016; Fried et al., 2011). A study involving intracortical microelectrode signals from the left motor cortex to assist various hand movements in a tetraplegic patient utilized a broader distribution of neural unit preferred directions across the 10D space to decode the motor cortex’s complex multidimensional capacity for BMI applications (Wodlinger et al., 2015) (Table 1). Prominent ERD of alpha and beta frequency were observed before (−1,000 ms) actual movement in the contralateral motor cortex during the executed movement in healthy adults and attempted movements in spinal cord injury patients (López-Larraz et al., 2014).

Yuan et al. found mu and beta rhythms to be predictive of the speed of hand movements that were performed or envisioned (Yuan et al., 2010). Moreover, Jochumsen et al. were able to decipher movement force and speed from MRCPs during attempted and actual grasping actions in stroke patients and healthy participants (Jochumsen et al., 2017). Similar experiments on patients and healthy participants have reported that beside velocity and position, it is also possible to discriminate different type of actions from non-invasive EEG (Hink et al., 1982; Ofner and Muller-Putz, 2012; Schwarz et al., 2017; Kobler et al., 2020; Mondini et al., 2024). In a novel experiment, intracranial EEG (iEEG) derived gamma power was used effectively as a BMI control signal for the task involving the reach and grasp movement (Fifer et al., 2014) (Table 1). Gu found that MRCPs contain a time-domain encoded representation of the velocity of executed wrist motions (Gu, 2009). According to Roosink and Zijdewind (2010), increased corticospinal excitability was observed during intricate successive finger-tapping imagery as compared to simple tapping (Roosink and Zijdewind, 2010) (Table 1). Further, complex hand movements such as sequential finger tapping, activate the SMA, PMC, superior parietal lobule (SPL), thalamus, and cerebellum to a greater extent than simple hand movements (Meister et al., 2005). In another study, activation of the same regions were found during complex spatiotemporal interlimb coordination (antiphase) compared to simple interlimb coordination (in-phase) (Debaere et al., 2004). In a recent study which investigated motor task complexity and dominance effect, it was found that movements performed during in-phase or anti-phase elicit distinct patterns of brain activity (Zhou et al., 2022) (Table 1). Complex arm movements involving combination of shoulder and elbow joints demonstrated specific muscle synergies for proximal and distal joints of the arm (Gritsenko et al., 2016). A study by Yeo et al. highlighted that during the execution of hand and shoulder movements, there was activation of distinct brain networks (Yeo et al., 2013). Slow cortical potentials (SCPs) were found to be predictive of trajectories of actual movement. Further, distinct brain regions were involved in hand and shoulder trajectories (Sosnik and Zur, 2020) (Table 1).

### 4.2 Effects of external assistance on the neural correlates of upper limb movements

The conversion of neural inputs into motion is important for the development of assistive devices to restore motor function in neurological disorders. During action execution, specific muscle groups are activated by neural signals that travel from the motor cortex through the spinal cord to the muscles, enabling the planned action to be carried out effectively (Guadagnoli and Lee, 2004).

Numerous techniques have been proposed by previous studies to decode joint angular velocities, movement directions, speed, locations, and intended movement trajectories (Bradberry et al., 2010; Ofner and Muller-Putz, 2012). Although EEG has been in use to decode these parameters, the poor correlation with actual or planned movement makes it difficult to provide precise and reliable control of prosthesis (Agashe et al., 2015; Ofner et al., 2017) (Table 2). Moreover, the neural signals that map motor commands and movements to specific regions of the PMC can vary depending on the position or orientation of the upper limb. This might imply that the re-interpretation of these parameters for BMI control is crucial whenever there is a shift in arm position.

**Table 2:** Effects of external assistance on the neural correlates of upper limb movements.

References	Sample size, tools used	Movement	Observations
Agashe et al. (2015)	5 healthy subjects, Mean age: 24.4 ± 5.2 years. EEG, cyber glove	Reach and grasping movements	Time-domain analysis revealed a correlation coefficient of 0.49 ± 0.02 between low-frequency (0.3–1 Hz) EEG bands and hand movement kinematics, with the highest reported coefficient being 0.59, indicating a strong predictive relationship between them. Early recruitment of contralateral frontal-central areas, followed by activation of central electrodes over primary sensorimotor areas, suggested a coordinated neural response related to movement execution.
Tacchino et al. (2017)	9 healthy subjects, Mean age 26.3 ± 1.9 years. EEG, EMG	Tasks with and without assistive glove	Activation started earlier (0.5 s) with the subject’s volitional contribution as compared to passive robotic assistance (1.5 s). Active-assisted training caused an earlier activation of the critical brain regions (i.e., sensorimotor cortex, PrMC, and SMA) and improved proprioceptive feedback.
Wilkins et al. (2017)	8 stroke survivors, Mean age: 63.5 ± 4 years. EEG, EMG-FES (Functional electrical stimulation)	Reaching and grasping movements	Brain activity associated with opening the affected hand was observed in the contralesional hemisphere. Chronic stroke survivors had both functional and structural cortical reconfiguration following device-assisted intervention.
Tang et al. (2023)	24 stroke patients with hemiplegia, Age: 18–80 years old. EEG	Bilateral upper limb robot-assisted training (BRT) for reaching and grasping movements	Quantitative EEG demonstrated increased connection in the primary motor cortex and supplementary motor area after BRT. BRT enhanced patients’ upper limb motor functions.
Cantillo-Negrete et al. (2021)	10 stroke patients, Mean age: 59.9 ± 12.8 years. EEG	ME and MI	Compared to conventional therapy, EEG based BCI showed substantially higher ERD in alpha band in central sagittal regions. For upper limb rehabilitation, a EEG based BCI system might be effective in fostering neuroplasticity.
Rayegani et al. (2014)	30 stroke survivors, Mean age: 45.1 ± 11.9 years. EEG, EMG	Hand function, specifically targeting the abductor pollicis brevis muscle	Neurofeedback training led to an increase in the spectral power density of the sensorimotor areas after 3.5 s of MI.

EEG, Electroencephalography; FES, Functional electrical stimulation; EMG, Electromyography; BCI, Brain computer interface; ME, Motor execution; MI, Motor imagery; PrMC, Premotor cortex; SMA, Supplementary motor area.

A previous study which explored brain activity using EEG and EMG signals found that active-assisted training caused an earlier activation of the critical brain regions such as the primary SMC, PrMC, and SMA. PMC encodes information related to action and sensing, which are relevant for clinical applications of BMIs (Serino et al., 2022; Mirabella and Lebedev, 2017; Sburlea and Muller-Putz, 2018). This region processes not only motor command but also sensory feedback and subjective states related to BMI-generated actions, underscoring the complexity of neural representation of agency involving multiple neural signals. The sense of agency, which reflects the feeling of control over one’s actions, is strengthened through such training, contributing to better motor learning and adaptation. It was also found that discrepancies between expected and actual sensory feedback can influence the sense of agency, emphasizing the role of sensory integration in the perception of control over actions (Haggard, 2017). Proprioceptive feedback was found to be augmented in the presence of the robotic aid, indicating that assistive technologies can enhance the engagement of neural circuits crucial for motor control (Tacchino et al., 2017) (Table 2). It is important to note that the neurological disorder-induced injury can trigger cortical plasticity, which might help compensate for impaired brain functions (Williamson et al., 2022). This enhancement of agency may further support cortical plasticity, promoting recovery. Further, disease severity, comorbid conditions, underlying pathophysiology of disease state, and age-related changes could determine the extent of recovery due to cortical plasticity. Hence, developing therapeutic interventions such as assistive training that could facilitate the recovery outcomes due to cortical plasticity is crucial (Marzola et al., 2023; Zhao et al., 2023).

Cortical neuroplasticity in chronic stroke survivors has been reported at both functional and structural levels following the use of external assistive devices for reach and grasp movements (Wilkins et al., 2017) (Table 2). Enhanced functional connectivity (FC) was observed in stroke patients due to robotic assistive therapy across various brain regions, including motor cortices, SMA, portions of the visuospatial system, cerebellum and association cortex. Importantly, improvements in upper-extremity function correlated with these FC alterations (Várkuti et al., 2013). In alignment with these findings, a study on bilateral upper limb robot-assisted training (BRT) for reaching and grasping movements found enhanced brain connectivity which correlated with motor recovery in stroke patients (Tang et al., 2023) (Table 2). Active robotic assistive therapy based on EEG was found to be more efficient than conventional therapy in stroke patients for upper-limb rehabilitation (Cantillo-Negrete et al., 2021) (Table 2). Assistive studies on hybrid BMIs have also shown efficiency in motor rehabilitation inducing cortical neuroplasticity (Pichiorri et al., 2023; Colamarino et al., 2023). Rohm et al. showed the restoration of both hand and elbow functions in tetraplegia when undergoing cue-based training using assistive devices designed on SMR-based BMI (Rohm et al., 2013). EEG neurofeedback enhances motor function in stroke patients by increasing the power spectral density of sensorimotor rhythm (Neuper et al., 2006).

Sensory feedback is important in motor control systems, particularly in feedback or closed-loop systems where the sensorimotor loop allows real-time adjustment of movements (Asan et al., 2022). Furthermore, the absence of sensory proprioception hampers the brain’s ability to predict movement and force, making tasks that require rapid directional changes difficult. This underscores the essential role of sensory feedback in motor control (Jayasinghe, 2019; Hehenberger et al., 2020). Through EMG biofeedback, stroke survivors enhance their motor recovery while fostering self-awareness and active engagement (Rayegani et al., 2014) (Table 2). Evidence suggests that the motor recovery due to assistive devices is marked by several key characteristics. Firstly, there is augmentation in the size of motor and sensory regions representing the impaired limb in the affected hemisphere. Secondly, there is an increase in activity and recruitment of motor networks located in unaffected regions. Thirdly, there is a gradual decrease in activity in the contralateral primary and secondary motor regions over time (Johnson, 2006). Neurofeedback uses real-time input to modify brain activity, hence facilitating functional improvement in motor rehabilitation and fostering neuroplasticity. Integrating EEG, EMG, and other physiological signals could improve the precision and reliability of BMI for motor rehabilitation (Wolpaw and Wolpaw, 2012).

### 4.3 Effects of changes in biomechanical characteristics on brain activity

Understanding how the human brain encodes biomechanical changes due to internally generated movement, externally assisted movement and environmental influence (e.g., gravity) is crucial for developing control systems for motor neuroprostheses and robotic arms. A recent study by Gaveau and Papaxanthis (2011) revealed that the CNS adjusts motor control in response to inertial and gravitational constraints (Gaveau and Papaxanthis, 2011) (Table 3). Research across electrophysiology and neuroimaging domains indicates distinct neural processes influencing different modes of action selection (Marini et al., 2019). Assistive wearables have been found to affect neuromuscular coordination (Gordon et al., 2013), motor complexity (Farris et al., 2013), and cognitive demands (Bequette et al., 2020).

**Table 3:** Effect of changes in biomechanical characteristics on brain activity.

Reference	Sample size, tools used	Movement	Observations
Gaveau and Papaxanthis (2011)	8 healthy subjects, Mean age: 24 ± 3. 3D Motion capture	Single-joint vertical arm movements (45° rotation around the shoulder joint) upwards and downwards	To reduce the energy expenditure of motions, brain uses gravity force to brake (upward direction) or initiate (downward direction) arm motion.
Le Seac’h and McIntyre (2007)	11 healthy subjects, Mean age: 28.1. 3D motion capture	Horizontal and vertical pointing movement	In the reclining position, information from subjects’ otoliths (which can potentially sense gravity’s direction) became less important to the movement. In the upright posture, saccules were extremely sensitive to gravity.
Namazi et al. (2018)	15 healthy subjects, Mean age: 27 ± 5. Fractal dimension (EEG signal complexity)	ME and MI: Elbow flexion/extension Forearm supination/pronation Hand open/close	More fractal dimensions were seen in the EEG signal during elbow flexion, forearm supination, and hand-open movements compared to the corresponding antagonistic actions.
Berret et al. (2008)	6 healthy subjects, Mean age: 29.6 ± 8.9. EMG, optoelectronic motion capture	Fast vertical arm movements	Motor planning incorporates both gravitational forces and inertial forces while minimizing an absolute-work-like cost. Periods of synchronized muscle inactivation were outcomes of the connectivity between the command circuits and the signals they interpret.
Teka et al. (2017)	A computational model for neural control of goal-directed reaching movements was developed.	Straight-line trajectory to a target position and a predefined bell-shaped velocity profile	Muscle velocities and muscle geometry determine the direction of cortical activation. Viscosity friction in the joints leads to the emergence of directional preference.
Pei et al. (2022)	10 healthy subjects, Mean age: 23.0 ± 3.1. EEG and cyber gloves.	Grasping of objects	Electrodes in the parietal and frontal regions showed a strong correlation with kinematic synergy.

EEG, Electroencephalography; EMG, Electromyograph; ME, Motor execution; MI, Motor imagery.

The brain optimizes vertical arm movements through dynamic planning, manipulating the neural representation of gravitational force to minimize muscle activation. This is in contrast to horizontal arm motions, where motor control is mostly dependent on kinematics. Specifically, in order to counteract gravity, the arm trajectory is asymmetrical due to the direction of movement (vertical versus horizontal movements) and the orientation of the body axis (Le Seac’h and McIntyre, 2007) (Table 3). In a study that investigated elbow flexion/extension, forearm supination/pronation, and hand open/close, a higher fractal dimension, i.e., increased EEG complexity was observed during elbow flexion, forearm supination, and hand-open movements compared to the corresponding antagonistic actions (Namazi et al., 2018) (Table 3).

Given that one of the primary goals of exoskeleton design is the “physical fit,” it is plausible to believe that there is a trade-off between the biomechanical and cognitive aspects of using them. For instance, a passive exoskeleton tailored for the upper limbs might minimize biomechanical loads on the shoulder and elbow joints, yet potentially elevate but increase neuropsychological demands cognitive, and sensory demands on the user. Therefore, the exact amount of this trade-off should be considered. Significant increments of cognitive demands by the exoskeleton can result in the alteration of muscle engagement patterns, muscle coactivity, and successive increases in spinal loading. As a result, the exoskeleton’s biomechanical benefits may decline or perhaps disappear entirely (Zhu et al., 2021). Previous reports suggest that the brain selects motions that minimize the total work of forces, which include gravitational and inertial forces which rely on movement direction and speed, respectively (Berret et al., 2008; Teka et al., 2017) (Table 3). A recent study found a strong correlation of EEG from parietal and frontal regions with the kinematic synergies of different grasping movements indicating the significant interaction between brain activity and movement dynamics to facilitate complex hand movements (Pei et al., 2022) (Table 3). EEG studies using multivariate pattern analysis have explored how the brain processes grasping movements over time. These studies examined neural activity as participants looked at objects and then performed either one-handed or two-handed grasps after being cued (Guo et al., 2021).

## 5 Concluding thoughts

Advancements in understanding the neural correlates of upper limb movements provide valuable insights for developing control mechanisms in assistive technologies. Here we systematically reviewed the recent developments in predicting the neural correlates of upper limb motor intention, the effects of external assistance on neural activity, and the influence of biomechanical changes on brain function. The findings emphasize significant progress in developing BMIs for upper limb rehabilitation while highlighting key challenges and future directions.

Translating neural signals into motion drives advancements in assistive technologies for neurological disorders. The motor system follows a hierarchical organization with specific brain regions assigned distinct roles. PPC and PrMC map peri-personal space. SMA/pre-SMA assesses the worthiness of goal-directed actions. LPFC integrates diverse information to choose appropriate goals, while PrMd, PMC, and PRR select actions to achieve these goals. A final evaluation occurs in a broader network, and PMC executes the movement by controlling muscle force and direction. Basal ganglia and cerebellum refine goal-directed movements alongside other regions of motor system. Further, basal ganglia coordinate goal-directed actions by exciting motor cortex (Parent, 2012) and prevent undesired actions by suppressing thalamus (Lanciego et al., 2012).

Despite these insights, the neural mechanisms generating motor commands, particularly for movement kinematics and kinetics in the sensorimotor cortex for BMI control, remain unclear. Nonetheless, existing literature may help identify the movement features best suited for BMI control. This paper provides a detailed review of EEG-based movement features relevant to BMI applications. To define motor intentions in BMI control, both MI and ME movement decoding are key factors (Demuru et al., 2013) that are reviewed in this study. MRCPs and ERD/S are studied to predict movement intention using EEG and the brain regions responsible are the PMC, SMA, and PrMC (Mirabella and Lebedev, 2017).

While EEG shows promise, overcoming challenges to achieve precise control for long-term use and addressing associated drawbacks remains essential. Closed-loop EEG-based BMIs could be a potential choice for long-term rehabilitation in patients. MI-based BMIs have been studied to enhance neural plasticity and motor function, particularly in stroke rehabilitation (Bhagat et al., 2020). However, decoding MI is still a challenging task due to inconsistent activation patterns in the brain. Advancements in machine learning, hold promise for advancing signal classification and assisting adaptive BMI systems (Yang et al., 2022).

The alterations in cortical representation due to arm position shifts or orientations necessitate continuous parameter recalibration. Continuous decoding of movement trajectories from EEG data show promise, but further research is needed. Variations in the predictive EEG activity patterns of movement intention and kinematics across individuals necessitate personalized tuning to enhance the efficiency of BMI (Lew et al., 2012).

Understanding how the brain adapts to biomechanical changes during movement is vital, with recent studies revealing modulatory brain activity in response to gravitational and inertial constraints, emphasizing the intricate interaction between neural processes and motor control (Gaveau and Papaxanthis, 2011). The brain adapts motor control strategies based on these biomechanical constraints to optimize movement execution of complex movements by activating broader networks, including the SMA, cerebellum, and parietal regions (Várkuti et al., 2013).

Assistive devices, alongside decreasing physical effort, can also increase cognitive demands due to the adaptation required by the participants (Bequette et al., 2020). This trade-off between the biomechanical and cognitive demands should be carefully studied to ensure the benefits of assistive devices outweigh the potential costs. Enhancing sensory feedback in BMI systems, such as providing real-time proprioceptive input, could significantly improve user experience and functional outcomes (Tacchino et al., 2017). Robotic-assisted therapy has been shown to enhance functional connectivity in motor-related brain regions (Cantillo-Negrete et al., 2021), correlating with improved motor function in stroke patients. Sensory integration and proprioceptive feedback are also enhanced with robotic assistance, improving the user’s sense of agency and motor performance (Haggard, 2017).

This review summarizes current literature on EEG-based neural features predictive of upper limb motor intention and kinematics, aiming to provide the research community with insights that support the development of EEG-based BMIs for motor rehabilitation. In addition, this review also summarizes current insights into how the brain encodes biomechanical changes from internally generated movement, external assistance, and environmental factors, and the effects of robotic assistance on neural activity and functional outcomes. EEG-based BMIs hold promise, yet challenges with signal variability and decoding accuracy persist. The extensive evidence for encoding multiple kinetic and kinematic parameters suggests that movement might best be understood as an integration of these variables, with more complex mechanistic models essential for accurately decoding motor behavior. Despite providing a comprehensive review, our study has certain limitations. This review focused exclusively on upper limb assistive devices in the adult population, thereby excluding research on lower limb exoskeletons and pediatric devices. Most of the included studies had small sample sizes and variations in study design, leading to potential heterogeneity in our study. Since we primarily examined neural correlates, we did not extensively cover BMI decoding techniques or control algorithms for assistive device functionality. Moreover, the lack of control groups, pre- vs. post-intervention comparisons, and follow-up data in certain studies posed a significant risk of bias. These limitations highlight the need for future studies with larger sample sizes, standardized methodologies, and rigorous bias assessments to strengthen the field’s evidence base. Future advancements in BMI systems for motor rehabilitation should assess decoding performance through these approaches, requiring the integration of multiple physiological signals, long-term performance stability, improved user engagement, and enhanced sensory feedback.

## References

[ref1] Agashe H. A. Paek A. Y. Zhang Y. Contreras-Vidal J. L. (2015). Global cortical activity predicts shape of hand during grasping. Front. Neurosci. 9:121. doi: 10.3389/fnins.2015.00121, PMID: 25914616 PMC4391035

[ref9001] Antonioni A. Raho E. M. Straudi S. Granieri E. Koch G. Fadiga L. (2024). The cerebellum and the Mirror Neuron System: a matter of inhibition? From neurophysiological evidence to neuromodulatory implications. A narrative review: Neurosci. Biobehav. Rev. 164:105830. doi: 10.1016/j.neubiorev.2024.105830 39069236

[ref9002] Asan A. S. McIntosh J. R. Carmel J. B. (2022). Targeting sensory and motor integration for recovery of movement after CNS injury. Front. Neurosci. 15:791824. doi: 10.3389/fnins.2021.791824 35126040 PMC8813971

[ref2] Bequette B. Norton A. Jones E. Stirling L. (2020). Physical and cognitive load effects due to a powered lower-body exoskeleton. Hum. Factors 62, 411–423. doi: 10.1177/0018720820907450, PMID: 32202434

[ref3] Berret B. Darlot C. Jean F. Pozzo T. Papaxanthis C. Gauthier J. P. (2008). The inactivation principle: mathematical solutions minimizing the absolute work and biological implications for the planning of arm movements. PLoS Comput. Biol. 4:e1000194. doi: 10.1371/journal.pcbi.1000194, PMID: 18949023 PMC2561290

[ref4] Bhagat N. A. Venkatakrishnan A. Abibullaev B. Artz E. J. Yozbatiran N. Blank A. A.. (2016). Design and optimization of an EEG-based brain machine Interface (BMI) to an upper-limb exoskeleton for stroke survivors. Front. Neurosci. 10:122. doi: 10.3389/fnins.2016.00122, PMID: 27065787 PMC4815250

[ref5] Bhagat N. A. Yozbatiran N. Sullivan J. L. Paranjape R. Losey C. Hernandez Z.. (2020). Neural activity modulations and motor recovery following brain-exoskeleton Interface mediated stroke rehabilitation. NeuroImage 28:102502. doi: 10.1016/j.nicl.2020.102502, PMID: 33395991 PMC7749405

[ref6] Bradberry T. J. Gentili R. J. Contreras-Vidal J. L. (2010). Reconstructing three-dimensional hand movements from noninvasive electroencephalographic signals. J. Neurosci. 30, 3432–3437. doi: 10.1523/JNEUROSCI.6107-09.2010, PMID: 20203202 PMC6634107

[ref7] Branco M. P. de Boer L. M. Ramsey N. F. Vansteensel M. J. (2019). Encoding of kinetic and kinematic movement parameters in the sensorimotor cortex: A brain-computer Interface perspective. Eur. J. Neurosci. 50, 2755–2772. doi: 10.1111/ejn.14342, PMID: 30633413 PMC6625947

[ref8] Caligiore D. Pezzulo G. Baldassarre G. Bostan A. C. Strick P. L. Doya K.. (2017). Consensus paper: towards a systems-level view of cerebellar function: the interplay between cerebellum, basal ganglia, and cortex. Cerebellum (London, England) 16, 203–229. doi: 10.1007/s12311-016-0763-3, PMID: 26873754 PMC5243918

[ref9] Cantillo-Negrete J. Carino-Escobar R. I. Carrillo-Mora P. Rodriguez-Barragan M. A. Hernandez-Arenas C. Quinzaños-Fresnedo J.. (2021). Brain-computer Interface coupled to a robotic hand orthosis for stroke patients’ Neurorehabilitation: A crossover feasibility study. Front. Hum. Neurosci. 15:656975. doi: 10.3389/fnhum.2021.656975, PMID: 34163342 PMC8215105

[ref10] Catrambone Vincenzo Greco Alberto Averta Giuseppe Bianchi Matteo Bicchi Antonio Scilingo Enzo Pasquale. (2018). “EEG complexity maps to characterise brain dynamics during upper limb motor imagery.” In 2018 40th Annual International Conference of the IEEE Engineering in Medicine and Biology Society (EMBC), 3060–3063. Honolulu, HI: IEEE. 10.1109/EMBC.2018.8512912 30441040

[ref11] Chavarriaga R. Sobolewski A. Millán Jdel R. (2014). Errare Machinale Est: the use of error-related potentials in brain-machine interfaces. Front. Neurosci. 8. doi: 10.3389/fnins.2014.00208, PMID: 25100937 PMC4106211

[ref12] Cisek P. Kalaska J. F. (2010). Neural mechanisms for interacting with a world full of action choices. Annu. Rev. Neurosci. 33, 269–298. doi: 10.1146/annurev.neuro.051508.135409, PMID: 20345247

[ref13] Colamarino E. Lorusso M. Pichiorri F. Toppi J. Tamburella F. Serratore G.. (2023). Discioser: unlocking recovery potential of arm sensorimotor functions after spinal cord injury by promoting activity-dependent brain plasticity by means of brain-computer interface technology: a randomized controlled trial to test efficacy. BMC Neurol. 23:414. doi: 10.1186/s12883-023-03442-w, PMID: 37990160 PMC10662594

[ref14] Cui H. Andersen R. A. (2007). Posterior parietal cortex encodes autonomously selected motor plans. Neuron 56, 552–559. doi: 10.1016/j.neuron.2007.09.031, PMID: 17988637 PMC2651089

[ref15] Debaere F. Wenderoth N. Sunaert S. Van Hecke P. Swinnen S. P. (2004). Cerebellar and premotor function in bimanual coordination: parametric neural responses to spatiotemporal complexity and cycling frequency. NeuroImage 21, 1416–1427. doi: 10.1016/j.neuroimage.2003.12.011, PMID: 15050567

[ref16] Deiber M.-P. Sallard E. Ludwig C. Ghezzi C. Barral J. Ibanez V. (2012). EEG alpha activity reflects motor preparation rather than the mode of action selection. Front. Integr. Neurosci. 6:59. doi: 10.3389/fnint.2012.00059, PMID: 22912607 PMC3418545

[ref17] Demuru M. Fara F. Fraschini M. (2013). Brain network analysis of EEG functional connectivity during imagery hand movements. J. Integr. Neurosci. 12, 441–447. doi: 10.1142/S021963521350026X, PMID: 24372064

[ref18] Diedrichsen J. Kornysheva K. (2015). Motor skill learning between selection and execution. Trends Cogn. Sci. 19, 227–233. doi: 10.1016/j.tics.2015.02.003, PMID: 25746123 PMC5617110

[ref19] Dokkum V. Liesjet E. H. Mottet D. Laffont I. Bonafé A. Menjot de Champfleur N.. (2017). Kinematics in the brain: unmasking motor control strategies? Exp. Brain Res. 235, 2639–2651. doi: 10.1007/s00221-017-4982-8, PMID: 28573311 PMC5550544

[ref9003] Dong Y. Wang S. Huang Q. Berg R. W. Li G. He J. (2023). Neural decoding for intracortical brain–computer interfaces. CBS, 4:0044. doi: 10.34133/cbsystems.0044 PMC10380541 37519930

[ref20] Edelman B. J. Meng J. Suma D. Zurn C. Nagarajan E. Baxter B. S.. (2019). Noninvasive neuroimaging enhances continuous neural tracking for robotic device control. Sci. Robot. 4:eaaw6844. doi: 10.1126/scirobotics.aaw6844, PMID: 31656937 PMC6814169

[ref21] Farris D. J. Robertson B. D. Sawicki G. S. (2013). Elastic ankle exoskeletons reduce soleus muscle force but not work in human hopping. J. Appl. Physiol. 115, 579–585. doi: 10.1152/japplphysiol.00253.2013, PMID: 23788578

[ref22] Fifer M. S. Hotson G. Wester B. A. McMullen D. P. Wang Y. Johannes M. S.. (2014). Simultaneous neural control of simple reaching and grasping with the modular prosthetic limb using intracranial EEG. IEEE Trans. Neural Syst. Rehabil. Eng. 22, 695–705. doi: 10.1109/TNSRE.2013.2286955, PMID: 24235276 PMC4030429

[ref23] Franchak J. M. van der Zalm D. J. Adolph K. E. (2010). Learning by doing: action performance facilitates affordance perception. Vis. Res. 50, 2758–2765. doi: 10.1016/j.visres.2010.09.019, PMID: 20858512 PMC3013505

[ref24] Fried I. Mukamel R. Kreiman G. (2011). Internally generated Preactivation of single neurons in human medial frontal cortex predicts volition. Neuron 69, 548–562. doi: 10.1016/j.neuron.2010.11.045, PMID: 21315264 PMC3052770

[ref25] Gandolfi M. Formaggio E. Geroin C. Storti S. F. Galazzo I. B. Bortolami M.. (2018). Quantification of upper limb motor recovery and EEG power changes after robot-assisted bilateral arm training in chronic stroke patients: A prospective pilot study. Neural Plast. 2018, 1–15. doi: 10.1155/2018/8105480, PMID: 29780410 PMC5892248

[ref26] Gaveau J. Papaxanthis C. (2011). The temporal structure of vertical arm movements. PLoS One 6:e22045. doi: 10.1371/journal.pone.0022045, PMID: 21765935 PMC3134452

[ref27] Genovesio A. Wise S. P. Passingham R. E. (2014). Prefrontal-parietal function: from foraging to foresight. Trends Cogn. Sci. 18, 72–81. doi: 10.1016/j.tics.2013.11.007, PMID: 24378542

[ref28] Gluud L. L. (2006). Bias in clinical intervention research. Am. J. Epidemiol. 163, 493–501. doi: 10.1093/aje/kwj069, PMID: 16443796

[ref29] Gordon K. E. Kinnaird C. R. Ferris D. P. (2013). Locomotor adaptation to a soleus EMG-controlled antagonistic exoskeleton. J. Neurophysiol. 109, 1804–1814. doi: 10.1152/jn.01128.2011, PMID: 23307949 PMC3628010

[ref30] Gritsenko V. Hardesty R. L. Boots M. T. Yakovenko S. (2016). Biomechanical constraints underlying motor primitives derived from the musculoskeletal anatomy of the human arm. PLoS One 11:e0164050. doi: 10.1371/journal.pone.0164050, PMID: 27736890 PMC5063279

[ref31] Gu Y. (2009). Off line identification of imagined speed of wrist movements in paralyzed ALS patients from single-trial EEG. Front. Neurosci. 3:62. doi: 10.3389/neuro.20.003.2009, PMID: 20582286 PMC2858603

[ref32] Guadagnoli M. A. Lee T. D. (2004). Challenge point: A framework for conceptualizing the effects of various practice conditions in motor learning. J. Mot. Behav. 36, 212–224. doi: 10.3200/JMBR.36.2.212-224, PMID: 15130871

[ref33] Guida P. Foffani G. Obeso I. (2023). The supplementary motor area and automatic cognitive control: lack of evidence from two Neuromodulation techniques. J. Cogn. Neurosci. 35, 439–451. doi: 10.1162/jocn_a_01954, PMID: 36603037

[ref34] Guo L. L. Oghli Y. S. Frost A. Niemeier M. (2021). Multivariate analysis of electrophysiological signals reveals the time course of precision grasps programs: evidence for nonhierarchical evolution of grasp control. J. Neurosci. Off. J. Soc. Neurosci. 41, 9210–9222. doi: 10.1523/JNEUROSCI.0992-21.2021, PMID: 34551938 PMC8570828

[ref35] Haggard P. (2017). Sense of agency in the human brain. Nat. Rev. Neurosci. 18, 196–207. doi: 10.1038/nrn.2017.14, PMID: 28251993

[ref36] Hehenberger L. Sburlea A. I. Müller-Putz G. R. (2020). Assessing the impact of vibrotactile kinaesthetic feedback on electroencephalographic signals in a center-out task. J. Neural Eng. 17:056032. doi: 10.1088/1741-2552/abb069, PMID: 33052887

[ref37] Hétu S. Grégoire M. Saimpont A. Coll M.-P. Eugène F. Michon P.-E.. (2013). The neural network of motor imagery: an ALE Meta-analysis. Neurosci. Biobehav. Rev. 37, 930–949. doi: 10.1016/j.neubiorev.2013.03.017, PMID: 23583615

[ref38] Higgins J. P. T. Altman D. G. Sterne J. A. C. (2008). Chapter 8: assessing risk of bias in included studies. In Cochrane handbook for systematic reviews of interventions, Cochrane. (Chichester) eds. Higgins J. P. T. Churchill R., Chandler J. Cumpston M. S..

[ref39] Hink R. F. Kohler H. Deecke L. Kornhuber H. H. (1982). Risk-taking and the human bereitschaftspotential. Electroencephalogr. Clin. Neurophysiol. 53, 361–373. doi: 10.1016/0013-4694(82)90002-5, PMID: 6175499

[ref40] Hochberg L. R. Serruya M. D. Friehs G. M. Mukand J. A. Saleh M. Caplan A. H.. (2006). Neuronal ensemble control of prosthetic devices by a human with tetraplegia. Nature 442, 164–171. doi: 10.1038/nature04970, PMID: 16838014

[ref41] Holmes N. P. Spence C. (2004). The body schema and the multisensory representation(s) of peripersonal space. Cogn. Process. 5, 94–105. doi: 10.1007/s10339-004-0013-3, PMID: 16467906 PMC1350799

[ref42] Hortal E. Planelles D. Resquin F. Climent J. M. Azorín J. M. Pons J. L. (2015). Using a brain-machine Interface to control a hybrid upper limb exoskeleton during rehabilitation of patients with neurological conditions. J. Neuroeng. Rehabil. 12:92. doi: 10.1186/s12984-015-0082-9, PMID: 26476869 PMC4609472

[ref9005] Jayasinghe S. A. L. (2019). The role of sensory stimulation on motor learning via action observation: a mini review. J. Neurophysiol. 121, 729–731. doi: 10.1152/jn.00747.2018 30517045

[ref43] Jochumsen M. Rovsing C. Rovsing H. Niazi I. K. Dremstrup K. Kamavuako E. N. (2017). Classification of hand grasp kinetics and types using movement-related cortical potentials and EEG rhythms. Comput. Intell. Neurosci. 2017, 1–8. doi: 10.1155/2017/7470864, PMID: 28951736 PMC5603104

[ref44] Johnson M. J. (2006). Recent trends in robot-assisted therapy environments to improve real-life functional performance after stroke. J. Neuroeng. Rehabil. 3:29. doi: 10.1186/1743-0003-3-29, PMID: 17176474 PMC1764881

[ref45] Jones L. A. Lederman S. J. (2006). Human Hand Function. USA: Oxford University Press.

[ref46] Klaes C. Westendorff S. Chakrabarti S. Gail A. (2011). Choosing goals, not rules: deciding among rule-based action plans. Neuron 70, 536–548. doi: 10.1016/j.neuron.2011.02.053, PMID: 21555078

[ref47] Kobler R. J. Kolesnichenko E. Sburlea A. I. Muller-Putz G. R. (2020). Distinct cortical networks for hand movement initiation and directional processing: an EEG study. NeuroImage 220:117076. doi: 10.1016/j.neuroimage.2020.117076, PMID: 32585349 PMC7573539

[ref48] Kornhuber H. H. Deecke L. (2016). Brain potential changes in voluntary and passive movements in humans: readiness potential and Reafferent potentials. Pflugers Arch. Eur. J. Physiol. 468, 1115–1124. doi: 10.1007/s00424-016-1852-3, PMID: 27392465

[ref49] Kraeutner S. N. MacKenzie L. A. Westwood D. A. Boe S. G. (2016). Characterizing skill acquisition through motor imagery with no prior physical practice. J. Exp. Psychol. Hum. Percept. Perform. 42, 257–265. doi: 10.1037/xhp0000148, PMID: 26389615

[ref50] Lanciego J. L. Luquin N. Obeso J. A. (2012). Functional neuroanatomy of the basal ganglia. Cold Spring Harb. Perspect. Med. 2:a009621. doi: 10.1101/cshperspect.a009621, PMID: 23071379 PMC3543080

[ref51] Le Seac’h A. B. McIntyre J. (2007). Multimodal reference frame for the planning of vertical arms movements. Neurosci. Lett. 423, 211–215. doi: 10.1016/j.neulet.2007.07.034, PMID: 17709199

[ref52] Lew E. Chavarriaga R. Silvoni S. Millán J. D. R. (2012). Detection of self-paced reaching movement intention from EEG signals. Front. Neuroeng. 5:13. doi: 10.3389/fneng.2012.00013, PMID: 23055968 PMC3458432

[ref53] Libet B. Gleason C. A. Wright E. W. Pearl D. K. (1983). Time of conscious intention to act in relation to onset of cerebral activity (readiness-potential). The unconscious initiation of a freely voluntary act. Brain J. Neurol. 106, 623–642. doi: 10.1093/brain/106.3.623, PMID: 6640273

[ref54] Lindner A. Iyer A. Kagan I. Andersen R. A. (2010). Human posterior parietal cortex plans where to reach and what to avoid. J. Neurosci. Off. J. Soc. Neurosci. 30, 11715–11725. doi: 10.1523/JNEUROSCI.2849-09.2010, PMID: 20810892 PMC2956133

[ref55] Lopes-Dias C. Sburlea A. I. Muller-Putz G. R. (2019). Online asynchronous decoding of error-related potentials during the continuous control of a robot. Sci. Rep. 9:17596. doi: 10.1038/s41598-019-54109-x, PMID: 31772232 PMC6879530

[ref56] López-Larraz E. Montesano L. Gil-Agudo Á. Minguez J. (2014). Continuous decoding of movement intention of upper limb self-initiated analytic movements from pre-movement EEG correlates. J. Neuroeng. Rehabil. 11:153. doi: 10.1186/1743-0003-11-153, PMID: 25398273 PMC4247645

[ref57] Lu M.-K. Arai N. Tsai C.-H. Ziemann U. (2012). Movement related cortical potentials of cued versus self-initiated movements: double dissociated modulation by dorsal premotor cortex versus supplementary motor area rTMS. Hum. Brain Mapp. 33, 824–839. doi: 10.1002/hbm.21248, PMID: 21425396 PMC6870267

[ref58] Makino H. Hwang E. J. Hedrick N. G. Komiyama T. (2016). Circuit mechanisms of sensorimotor learning. Neuron 92, 705–721. doi: 10.1016/j.neuron.2016.10.029, PMID: 27883902 PMC5131723

[ref59] Marini F. Zenzeri J. Pippo V. Morasso P. Campus C. (2019). Neural correlates of proprioceptive upper limb position matching. Hum. Brain Mapp. 40, 4813–4826. doi: 10.1002/hbm.24739, PMID: 31348604 PMC6865654

[ref9007] Marzola P. Melzer T. Pavesi E. Gil-Mohapel J. Brocardo P. S. (2023). Exploring the role of neuroplasticity in development, aging, and neurodegeneration. Brain Sci. 13:1610. doi: 10.3390/brainsci13121610 38137058 PMC10741468

[ref60] Meister I. Krings T. Foltys H. Boroojerdi B. Müller M. Töpper R.. (2005). Effects of long-term practice and task complexity in musicians and nonmusicians performing simple and complex motor tasks: implications for cortical motor organization. Hum. Brain Mapp. 25, 345–352. doi: 10.1002/hbm.20112, PMID: 15852385 PMC6871746

[ref61] Mirabella G. (2014). Should I stay or should I go? Conceptual underpinnings of goal-directed actions. Front. Syst. Neurosci. 8:206. doi: 10.3389/fnsys.2014.00206, PMID: 25404898 PMC4217496

[ref62] Mirabella G. (2021). “Does the power to suppress an action make us ‘free’?”. In: Opris I. A. Lebedev M. F. Casanova M.. (eds) Modern approaches to augmentation of brain function. Contemporary clinical neuroscience. Springer, Cham.

[ref63] Mirabella G. Lebedev M. А. (2017). Interfacing to the brain’s motor decisions. J. Neurophysiol. 117, 1305–1319. doi: 10.1152/jn.00051.2016, PMID: 28003406 PMC5350270

[ref64] Mizuguchi N. Nakamura M. Kanosue K. (2017). Task-dependent engagements of the primary visual cortex during kinesthetic and visual motor imagery. Neurosci. Lett. 636, 108–112. doi: 10.1016/j.neulet.2016.10.064, PMID: 27826015

[ref65] Mizuguchi N. Nakata H. Kanosue K. (2016). The right Temporoparietal junction encodes efforts of others during action observation. Sci. Rep. 6:30274. doi: 10.1038/srep30274, PMID: 27458025 PMC4960610

[ref66] Mondini V. Sburlea A. I. Müller-Putz G. R. (2024). Towards unlocking motor control in spinal cord injured by applying an online EEG-based framework to decode motor intention, trajectory and error processing. Sci. Rep. 14:4714. doi: 10.1038/s41598-024-55413-x, PMID: 38413782 PMC10899181

[ref67] Muller-Putz G. R. Kobler R. J. Pereira J. Lopes-Dias C. Hehenberger L. Mondini V.. (2022). Feel your reach: an EEG-based framework to continuously detect goal-directed movements and error processing to gate kinesthetic feedback informed artificial arm control. Front. Hum. Neurosci. 16:841312. doi: 10.3389/fnhum.2022.841312, PMID: 35360289 PMC8961864

[ref68] Namazi H. Ala T. S. Kulish V. (2018). Decoding of upper limb movement by fractal analysis of electroencephalogram (EEG) signal. Fractals 26:1850081. doi: 10.1142/S0218348X18500810

[ref69] Neuper C. Muller-Putz G. R. Scherer R. Pfurtscheller G. (2006). Motor imagery and EEG-based control of spelling devices and Neuroprostheses. Prog. Brain Res. 159, 393–409. doi: 10.1016/S0079-6123(06)59025-9, PMID: 17071244

[ref70] Ofner P. Muller-Putz G. R. (2012). “Decoding of velocities and positions of 3D arm movement from EEG.” In 2012 Annual International Conference of the IEEE Engineering in Medicine and Biology Society, 6406–6409. San Diego, CA: IEEE. 10.1109/EMBC.2012.6347460 23367395

[ref71] Ofner P. Muller-Putz G. R. (2015). Using a noninvasive decoding method to classify rhythmic movement imaginations of the arm in two Planes. IEEE Trans. Biomed. Eng. 62, 972–981. doi: 10.1109/TBME.2014.2377023, PMID: 25494495

[ref72] Ofner P. Schwarz A. Pereira J. Muller-Putz G. R. (2017). Upper limb movements can bedecoded from the time-domain of low-frequency EEG. PloS one 12:e0182578. doi: 10.1371/journal.pone.0182578 28797109 PMC5552335

[ref73] Ofner P. Schwarz A. Pereira J. Wyss D. Wildburger R. Muller-Putz G. R. (2019). Attempted arm and hand movements can be decoded from low-frequency EEG from persons with spinal cord injury. Sci. Rep. 9:7134. doi: 10.1038/s41598-019-43594-9, PMID: 31073142 PMC6509331

[ref74] Omedes J. Schwarz A. Muller-Putz G. R. Montesano L. (2018). Factors that affect error potentials during a grasping task: toward a hybrid natural movement decoding BCI. J. Neural Eng. 15:046023. doi: 10.1088/1741-2552/aac1a1, PMID: 29714718

[ref75] Ottenhoff M. C. Verwoert M. Goulis S. Colon A. J. Wagner L. Tousseyn S.. (2023). Decoding executed and imagined grasping movements from distributed non-motor brain areas using a Riemannian decoder. Front. Neurosci. 17:1283491. doi: 10.3389/fnins.2023.1283491, PMID: 38075279 PMC10701391

[ref9006] Page M. J. McKenzie J. E. Bossuyt P. M. Boutron I. Hoffmann T. C. Mulrow C. D. (2021). The PRISMA 2020 statement: an updated guideline for reporting systematic reviews. BMJ Clin. Res. 372:n71. doi: 10.1136/bmj.n71 PMC8005924 33782057

[ref76] Parent A. (2012). The history of the basal ganglia: the contribution of Karl Friedrich Burdach. Neurosci. Med. 3, 374–379. doi: 10.4236/nm.2012.34046

[ref77] Pei D. Olikkal P. Adali T. Vinjamuri R. (2022). Reconstructing synergy-based hand grasp kinematics from electroencephalographic signals. Sensors 22:5349. doi: 10.3390/s22145349, PMID: 35891029 PMC9318424

[ref78] Pereira J. Ofner P. Schwarz A. Sburlea A. I. Muller-Putz G. R. (2017). EEG neural correlates of goal-directed movement intention. NeuroImage 149, 129–140. doi: 10.1016/j.neuroimage.2017.01.030 28131888 PMC5387183

[ref79] Pfurtscheller G. Aranibar A. (1979). Evaluation of event-related desynchronization (ERD) preceding and following voluntary self-paced movement. Electroencephalogr. Clin. Neurophysiol. 46, 138–146. doi: 10.1016/0013-4694(79)90063-4, PMID: 86421

[ref80] Pichiorri F. Toppi J. de Seta V. Colamarino E. Masciullo M. Tamburella F.. (2023). Exploring high-density corticomuscular networks after stroke to enable a hybrid brain-computer Interface for hand motor rehabilitation. J. Neuroeng. Rehabil. 20:5. doi: 10.1186/s12984-023-01127-6, PMID: 36639665 PMC9840279

[ref81] Pulferer H. S. Ásgeirsdóttir B. Mondini V. Sburlea A. I. Muller-Putz G. R. (2022). Continuous 2D trajectory decoding from attempted movement: across-session performance in able-bodied and feasibility in a spinal cord injured participant. J. Neural Eng. 19, 036005–032552. doi: 10.1088/1741-2552/ac689f 35443233

[ref82] Rayegani S. M. Raeissadat S. A. Sedighipour L. Mohammad Rezazadeh I. Bahrami M. H. Eliaspour D.. (2014). Effect of Neurofeedback and Electromyographic-biofeedback therapy on improving hand function in stroke patients. Top. Stroke Rehabil. 21, 137–151. doi: 10.1310/tsr2102-137, PMID: 24710974

[ref83] Rocha G. S. Freire M. A. M. Britto A. M. Paiva K. M. Oliveira R. F. Fonseca I.. (2023). Basal ganglia for beginners: the basic concepts you need to know and their role in movement control. Front. Syst. Neurosci. 17:1242929. doi: 10.3389/fnsys.2023.1242929, PMID: 37600831 PMC10435282

[ref84] Rohm M. Schneiders M. Müller C. Kreilinger A. Kaiser V. Muller-Putz G. R.. (2013). Hybrid brain–computer interfaces and hybrid Neuroprostheses for restoration of upper limb functions in individuals with high-level spinal cord injury. Artifi. Intell. Med 59, 133–142. doi: 10.1016/j.artmed.2013.07.004 24064256

[ref85] Roosink M. Zijdewind I. (2010). Corticospinal excitability during observation and imagery of simple and complex hand tasks: implications for motor rehabilitation. Behav. Brain Res. 213, 35–41. doi: 10.1016/j.bbr.2010.04.027, PMID: 20433871

[ref86] Rossini P. M. Ferilli M. A. N. Ferreri F. (2012). Cortical plasticity and brain computer Interface. Eur. J. Phys. Rehabil. Med. 48, 307–312. 22614891

[ref87] Rupp R. Gerner H. J. (2004). Neuroprosthetics of the Upper Extremity – Clinical Application in Spinal Cord Injury and Future Perspectives / Neuroprothetik Der Oberen Extremität – Klinische Einsatzmöglichkeiten Bei Querschnittlähmung Und Perspektiven Für Die Zukunft. Biomed. Eng. 49, 93–98. doi: 10.1515/BMT.2004.019, PMID: 15171589

[ref88] Rupp R. Rohm M. Schneiders M. Kreilinger A. Muller-Putz G. R. (2015). Functional rehabilitation of the paralyzed upper extremity after spinal cord injury by noninvasive hybrid Neuroprostheses. Proc. IEEE 103, 954–968. doi: 10.1109/JPROC.2015.2395253

[ref89] Sambhav R. Jena S. Chatterjee A. Bhasin S. Santapuri S. Kumar L.. (2022). An integrated dynamic closed loop simulation platform for elbow flexion augmentation using an upper limb exosuit model. Front. Robot. AI 9:768841. doi: 10.3389/frobt.2022.768841, PMID: 35368436 PMC8967966

[ref90] Sburlea A. I. Muller-Putz G. R. (2018). Exploring representations of human grasping in neural, muscle and kinematic signals. Sci. Rep. 8:16669. doi: 10.1038/s41598-018-35018-x, PMID: 30420724 PMC6232146

[ref91] Sburlea A. I. Wilding M. Muller-Putz G. (2021). Disentangling human grasping type from the object’s intrinsic properties using low-frequency EEG signals. Neuroimage 1:100012. doi: 10.1016/j.ynirp.2021.100012, PMID: 39843627

[ref92] Schultze-Kraft M. Birman D. Rusconi M. Allefeld C. Görgen K. Dähne S.. (2016). The point of no return in vetoing self-initiated movements. Proc. Nat. Acad. Sci. United States of America 113, 1080–1085. doi: 10.1073/pnas.1513569112, PMID: 26668390 PMC4743787

[ref93] Schünemann H. J. Cuello C. Akl E. A. Mustafa R. A. Meerpohl J. J. Thayer K.. (2018). GRADE guidelines: 18. How ROBINS-I and other tools to assess risk of bias in nonrandomized studies should be used to rate the certainty of a body of evidence. J. Clin. Epidemiol. 111, 105–114. doi: 10.1016/j.jclinepi.2018.01.012, PMID: 29432858 PMC6692166

[ref9011] Schwarz A. Ofner P. Pereira J. Sburlea A. I. Müller-Putz G. R. (2017). Decoding natural reach-and-grasp actions from human EEG. J Neural Eng. 15:016005. doi: 10.1088/1741-2552/aa8911 28853420

[ref94] Scott S. H. (2016). A functional taxonomy of bottom-up sensory feedback processing for motor actions. Trends Neurosci. 39, 512–526. doi: 10.1016/j.tins.2016.06.001, PMID: 27378546

[ref95] Serino A. Bockbrader M. Bertoni T. Iv S. C. Solcà M. Dunlap C.. (2022). Sense of Agency for Intracortical Brain–Machine Interfaces. Nat. Hum. Behav. 6, 565–578. doi: 10.1038/s41562-021-01233-2 35046522

[ref96] Singh R. E. Iqbal K. White G. Hutchinson T. E. (2018). A systematic review on muscle synergies: from building blocks of motor behavior to a Neurorehabilitation tool. Appl. Bion. Biomech. 2018, 3615368–3615315. doi: 10.1155/2018/3615368, PMID: 29849756 PMC5937559

[ref97] Sosnik R. Zur O. B. (2020). Reconstruction of hand, elbow and shoulder actual and imagined trajectories in 3D space using EEG slow cortical potentials. J. Neural Eng. 17:016065. doi: 10.1088/1741-2552/ab59a7, PMID: 31747655

[ref98] Spuler M. Niethammer C. (2015). Error-related potentials during continuous feedback: using EEG to detect errors of different type and severity. Front. Hum. Neurosci. 9:155. doi: 10.3389/fnhum.2015.00155, PMID: 25859204 PMC4374466

[ref99] Sterne J. A. Hernán M. A. Reeves B. C. Savović J. Berkman N. D. Viswanathan M.. (2016). ROBINS-I: a tool for assessing risk of bias in non-randomised studies of interventions. BMJ 355:i4919. doi: 10.1136/bmj.i4919, PMID: 27733354 PMC5062054

[ref100] Tacchino G. Gandolla M. Coelli S. Barbieri R. Pedrocchi A. Bianchi A. M. (2017). EEG analysis during active and assisted repetitive movements: evidence for differences in neural engagement. IEEE Trans. Neural Syst. Rehabil. Eng. 25, 761–771. doi: 10.1109/TNSRE.2016.2597157, PMID: 27529874

[ref101] Tang Z. Sun S. Zhang S. Chen Y. Li C. Chen S. (2016). A brain-machine Interface based on ERD/ERS for an upper-limb exoskeleton control. Sensors 16:2050. doi: 10.3390/s16122050, PMID: 27918413 PMC5191031

[ref102] Tang C. Zhou T. Zhang Y. Yuan R. Zhao X. Yin R.. (2023). Bilateral upper limb robot-assisted rehabilitation improves upper limb motor function in stroke patients: A study based on quantitative EEG. Eur. J. Med. Res. 28:603. doi: 10.1186/s40001-023-01565-x, PMID: 38115157 PMC10729331

[ref103] Teka W. W. Hamade K. C. Barnett W. H. Kim T. Markin S. N. Rybak I. A.. (2017). From the motor cortex to the movement and Back again. PLoS One 12:e0179288. doi: 10.1371/journal.pone.0179288, PMID: 28632736 PMC5478113

[ref104] Ullsperger M. Danielmeier C. Jocham G. (2014). Neurophysiology of performance monitoring and adaptive behavior. Physiol. Rev. 94, 35–79. doi: 10.1152/physrev.00041.2012, PMID: 24382883

[ref105] Varghese R. J. Freer D. Deligianni F. Liu J. Yang G.-Z. Tong R. (2018). “Wearable robotics for upper-limb rehabilitation and assistance: a review of the state-of-the-art challenges and future research,” In Wearable Technology in Medicine and Health Care, (Elsevier) 23–69. doi: 10.1016/B978-0-12-811810-8.00003-8

[ref106] Várkuti B. Guan C. Pan Y. Phua K. S. Ang K. K. Kuah C. W. K.. (2013). “Resting state changes in functional connectivity correlate with movement recovery for BCI and robot-assisted upper-extremity training after stroke,” in Neurorehabil. Neural Repair 27, 53–62. doi: 10.1177/1545968312445910, PMID: 22645108

[ref107] Wang J. Bi L. Fei W. (2023). EEG-based motor BCIs for upper limb movement: current techniques and future insights. IEEE Trans. Neural Syst. Rehabil. Eng. 31, 4413–4427. doi: 10.1109/TNSRE.2023.3330500, PMID: 37930905

[ref109] Wilkins K. B. Owen M. Ingo C. Carmona C. Dewald J. P. A. Yao J. (2017). Neural plasticity in moderate to severe chronic stroke following a device-assisted task-specific arm/hand intervention. Front. Neurol. 8:284. doi: 10.3389/fneur.2017.00284, PMID: 28659863 PMC5469871

[ref9009] Williamson J. N. Sikora W. A. James S. A. Parmar N. J. Lepak L. V. Cheema C. F.. (2022). Cortical reorganization of early somatosensory processing in hemiparetic stroke. J Clin. Med. 11:6449. doi: 10.3390/jcm11216449 36362680 PMC9654771

[ref110] Wodlinger B. Downey J. E. Tyler-Kabara E. C. Schwartz A. B. Boninger M. L. Collinger J. L. (2015). Ten-dimensional anthropomorphic arm control in a human brain−machine Interface: difficulties, solutions, and limitations. J. Neural Eng. 12:016011. doi: 10.1088/1741-2560/12/1/016011, PMID: 25514320

[ref111] Wolpaw J. R. Wolpaw E. W. (2012). Brain-computer interfaces: something new under the sun. Brain-computer interfaces: principles and practice, 14, 3–12. doi: 10.1093/acprof:oso/9780195388855.001.0001

[ref112] Wu Z. Reddy R. Pan G. Zheng N. Verschure P. F. M. J. Zhang Q.. (2013). The convergence of machine and biological intelligence. IEEE Intell. Syst. 28, 28–43. doi: 10.1109/MIS.2013.137

[ref113] Xu R. Jiang N. Lin C. Mrachacz-Kersting N. Dremstrup K. Farina D. (2014). Enhanced low-latency detection of motor intention from EEG for closed-loop brain-computer interface applications. I.E.E.E. Trans. Biomed. Eng. 61, 288–296. doi: 10.1109/TBME.2013.2294203, PMID: 24448593

[ref114] Yang B. Ma J. Qiu W. Zhang J. Wang X. (2022). The unilateral upper limb classification from fMRI-weighted EEG signals using convolutional neural network. Biomed. Signal Proc. Control 78:103855. doi: 10.1016/j.bspc.2022.103855, PMID: 39843627

[ref115] Yeo S. S. Chang P.-H. Jang S. H. (2013). The cortical activation differences between proximal and distal joint movements of the upper extremities: A functional NIRS study. NeuroRehabilitation 32, 861–866. doi: 10.3233/NRE-130910, PMID: 23867412

[ref116] Yong X. Menon C. (2015). EEG classification of different imaginary movements within the same limb. PLoS One 10:e0121896. doi: 10.1371/journal.pone.0121896, PMID: 25830611 PMC4382224

[ref117] Yuan H. Perdoni C. He B. (2010). Relationship between speed and EEG activity during imagined and executed hand movements. J. Neural Eng. 7:026001. doi: 10.1088/1741-2560/7/2/026001, PMID: 20168002 PMC3036745

[ref118] Zhao K. Zhang Z. Wen H. Liu B. Li J. d’Avella A.. (2023). Muscle synergies for evaluating upper limb in clinical applications: A systematic review. Heliyon 9:e16202. doi: 10.1016/j.heliyon.2023.e16202, PMID: 37215841 PMC10199229

[ref119] Zhou G. Chen Y. Wang X. Wei H. Huang Q. Li L. (2022). The correlations between kinematic profiles and cerebral hemodynamics suggest changes of motor coordination in single and bilateral finger movement. Front. Hum. Neurosci. 16:957364. doi: 10.3389/fnhum.2022.957364, PMID: 36061505 PMC9433536

[ref120] Zhu Y. Weston E. B. Mehta R. K. Marras W. S. (2021). Neural and biomechanical tradeoffs associated with human-exoskeleton interactions. Appl. Ergon. 96:103494. doi: 10.1016/j.apergo.2021.103494, PMID: 34126572

