# Supplementary figures and images for: Decoding the brain-machine interaction for upper limb assistive technologies: advances and challenges
Source: Front Hum Neurosci. 2025 Feb 6;19:1532783. doi: 10.3389/fnhum.2025.1532783 (PMC11839673; doi:10.3389/fnhum.2025.1532783)

**
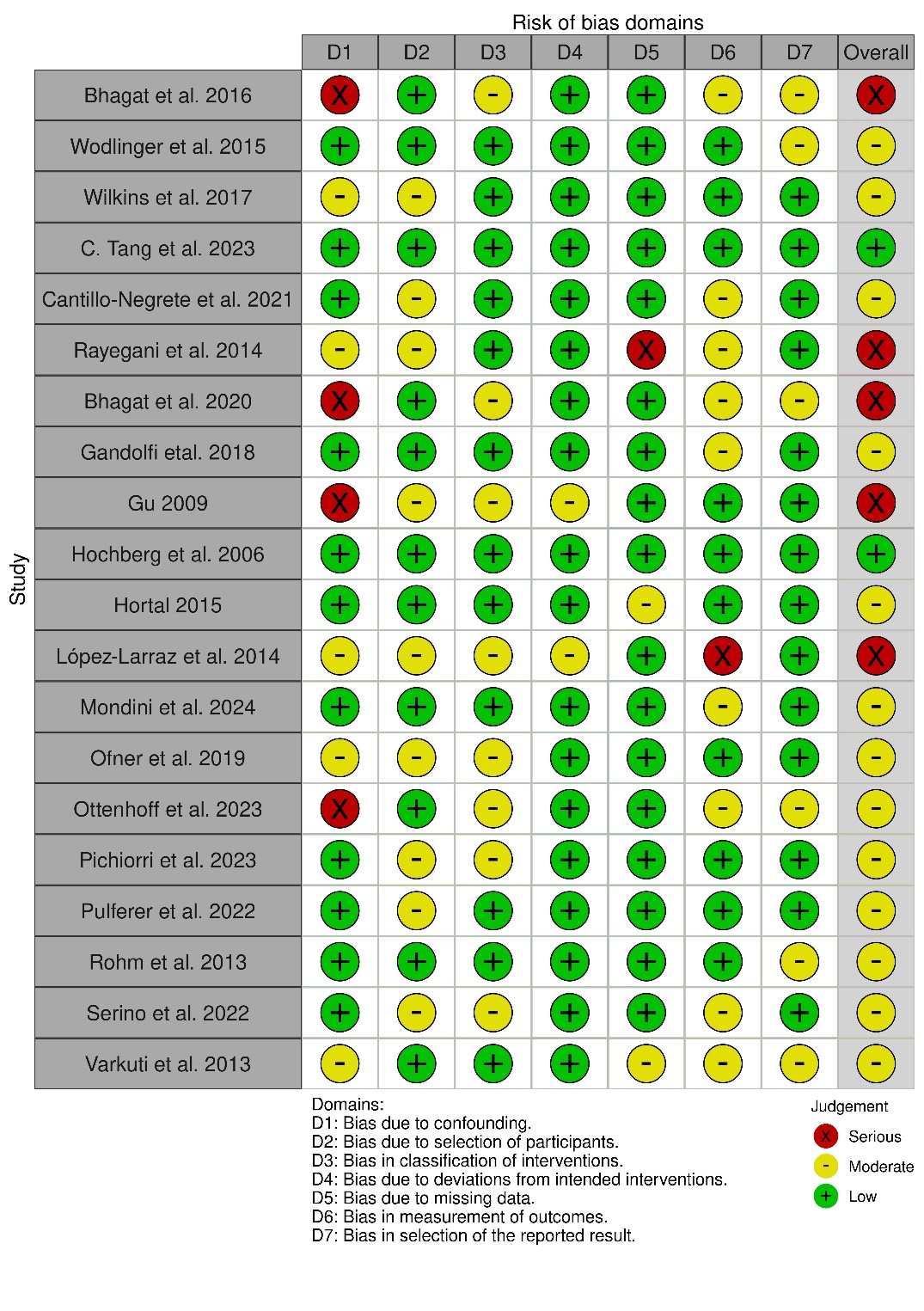
Appendix 1**

Appendix 1: Traffic light plot for risk of bias of included clinical studies

Supplement: Supplementary file 1 [file Data_Sheet_1.docx]
